# Quality of care in an era of global challenges: a transformational vision for WHO European Region and beyond

**DOI:** 10.1093/eurpub/ckaf204

**Published:** 2025-11-05

**Authors:** Válter R Fonseca, Damir Ivanković, Blerta Maliqi, Niek Klazinga, Stefan Larsson, Ernst J Kuipers, Christos Triantafyllou, Jennifer Hall, Lilian Vildiridi, Govin Permanand, Natasha Azzopardi Muscat, João Breda

**Affiliations:** WHO Athens Quality of Care and Patient Safety Office, World Health Organization Regional Office for Europe, Athens, Greece; WHO Athens Quality of Care and Patient Safety Office, World Health Organization Regional Office for Europe, Athens, Greece; World Health Organization, Geneva, Switzerland; Department of Public and Occupational Health, Amsterdam UMC Location University of Amsterdam, Amsterdam, The Netherlands; The International Consortium for Health Outcomes Measurement, Boston, MA, United States; Lee Kong Chian School of Medicine, Nanyang Technological University, Singapore, Singapore; WHO Athens Quality of Care and Patient Safety Office, World Health Organization Regional Office for Europe, Athens, Greece; WHO Athens Quality of Care and Patient Safety Office, World Health Organization Regional Office for Europe, Athens, Greece; Ministry of Health, Athens, Greece; World Health Organization Regional Office for Europe, Copenhagen, Denmark; World Health Organization Regional Office for Europe, Copenhagen, Denmark; WHO Athens Quality of Care and Patient Safety Office, World Health Organization Regional Office for Europe, Athens, Greece

## Abstract

Health systems today face overlapping pressures—from demographic shifts, workforce shortages, climate change, and geopolitical and economic instability. This strains their ability to deliver effective and equitable care and erodes public trust. Traditional approaches to quality of care, often focused on service volumes or process compliance, are proving insufficient to address these system-wide challenges. In response, this paper proposes a transformational vision for quality of care that moves beyond traditional models. This vision is rooted in two interconnected pillars. First, a focus on outcomes that truly matter to people and populations, prioritizing health and well-being over service volume. The second pillar is a whole-systems perspective that embeds quality across all levels of governance, policy, and financing. This transformation is made possible through three key enablers. First, an empowered workforce and accountable leadership are needed to drive change. Second, data must be used transparently to build trust and guide results-focused work. Finally, innovative solutions and tools must enhance quality and be aligned with equity. Drawing on practical implementation examples, this paper outlines a roadmap for system-wide alignment of health systems—to rebuild trust, improve resource use, and advance health equity. This makes quality a lasting foundation for resilient, sustainable, and equitable healthcare.

## Introduction

### The need for an improved approach to quality

Interconnected global challenges are putting health systems under increasing strain, testing their ability to provide high-quality care that is safe, effective, people-centered, timely, equitable, integrated, and efficient [1]. Geopolitical and economic instabilities, migration and demographic shifts, workforce shortages, climate change, commercial determinants of health and evolving disease burdens are reshaping healthcare priorities [2]. The COVID-19 pandemic exposed critical vulnerabilities in health system governance, workforce resilience, and digital preparedness [3]. Rising healthcare costs and economic volatility strain budgets and emphasize the need to balance cost-efficiency, sustainability, and quality [4]. Trust in health systems is eroding due to rising misinformation and disinformation, coupled with widening health disparities [5].

Decades of quality of care scholarship, from Donabedian’s structure–process–outcome model to the Institute of Medicine’s landmark “Crossing the Quality Chasm” report, have broadened quality to include safety, effectiveness, efficiency, timeliness, equity, integration, and people-centeredness [6–8]. Quality of care is often considered as the degree of standardization of clinical processes and compliance with guidelines. While essential, these are insufficient to bring coherence and purpose across fragmented health systems. Traditional quality models, focused on structural indicators or service volumes, often fall short in improving real-world health outcomes. This additionally leads to increased administrative burdens, fragmented care delivery, limited patient-centered innovation, and rising inequities, ultimately hindering health system’s transformation capacity and trust [5]. The increasing complexity of today’s health systems requires moving beyond these foundations, toward more integrated and adaptive approaches.

Calls for action on quality of care have previously been made by major global reports. In 2018, the joint World Health Organization (WHO), World Bank and Organization for Economic Co-operation and Development (OECD) published the “Delivering quality health services: A global imperative” report. It underscored that poor-quality care is a bigger barrier to reducing mortality than lack of access [9]. In the same year, the Lancet Global Health Commission on High Quality Health Systems called for health systems to focus on competent care and positive user experience [10]. Both reports position quality as central to reaching Sustainable Development Goals (SDGs) and a cornerstone of Universal Health Coverage (UHC). In 2017, OECD highlighted that wasteful practices in healthcare, which account for 20%–40% of total costs, can be reduced by prioritizing patient outcomes and quality-driven innovation [11]. An all-level focus on quality provides a common purpose that connects otherwise siloed efforts in governance, digitalization, financing, and workforce development. Systems that invest in quality reduce waste and make better use of limited workforce. In an era of budgetary constraints and mounting health worker strain, such quality focus becomes a lever to do more with less [12, 13].

Almost a decade later, the WHO Regional Office for Europe’s 2024 “Taking the pulse of quality of care and patient safety in the WHO European Region” report still identified significant disparities in preventable mortality, patient safety, access to care, financial protection, and digital health adoption across Regional Member States [14].

Quality of care cannot be separated from the quality of health systems as a whole. In this context, quality must be seen not as a technical goal but as a fundamental principle of health system effectiveness, efficiency, sustainability, resilience and equity.

Combining and evolving existing evidence and quality of care concepts, we show that a transformation toward a systems-thinking, outcome-driven, and patient-centered vision of quality is needed—one that fosters continuous quality improvement, staff engagement, and long-term system sustainability. To translate this into action, we propose fundamental pillars and enablers.

### A transformational vision of quality

In this paper, we frame this transformational vision through a whole-systems approach that strengthens quality-focused governance and ensures sustainability. This is not a completely new agenda but rather an evolution of existing commitments. The vision aligns with the “Framework for Resilient and Sustainable Health Systems in the WHO European Region” and the upcoming WHO Second European Program of Work 2026–30 (EPW2) [15, 16]. EPW2 identifies key health priorities, such as health security, non-communicable diseases, mental health, healthy aging, and climate resilience. It also recognizes the relevance of embedding data-driven outcome measurements—whether through primary care, prevention, remote care, or integrated services—to enhance transparency, identify disparities, and guide policy decisions and actions.

A transformational vision for quality drives health systems to improve performance and deliver better outcomes that matter to people—safer care, more equitable access, and improved health and well-being. It builds trust by making systems more transparent and accountable—for example, through public reporting of outcomes and participatory governance, which includes both patient and health workforce involvement in co-designing policies—while encouraging innovation grounded in real-world impact and human rights. This evolving vision is shaped by two interconnected pillars:

- Outcomes that matter—Defining and measuring quality based on its actual impact on people’s health and well-being, rather than on the volume of services delivered or adherence to processes.
- Systems perspective to quality—Embedding quality, as defined above, at all levels, from governance and workforce to financing and digital transformation.

These pillars are supported by three key enablers:

- People, leadership and a drive for change—Strengthening workforce and community engagement, leadership development to drive system quality and building institutional resilience.
- Data and transparency—Utilizing data systems for evidence-based decisions and fostering accountability and trust.
- Innovative solutions and tools—Promoting relevant innovation that enhances outcomes that matter, while focusing on safety, effectiveness, equity, people-centeredness, and efficiency.

This approach aims to strengthen quality of care, aligning measurement with patient-centered outcomes, embedding quality across all system levels, and fostering innovation that build trust, resilience and sustainability. The vision is enabled by empowered healthcare professionals, patients and communities, transparent and actionable data, and innovation that advances equity. In an era of multiple challenges, it proposes a renewed focus on quality—one that helps (re)build adaptive and trustworthy health systems.

## Pillars of quality transformation

### Outcomes that matter

While adherence to standards remains important for day-to-day operations, the ultimate goal of high-quality healthcare is to deliver people-centered care that is both effective and efficient. This focus moves beyond the service-level lens of health care and reflects a broader shift toward a whole-system, value-increasing perspective—one that integrates equity, solidarity, transparency, participation, and population health with financial sustainability [17, 18]. This positions quality and outcomes as central components of system’s performance. In this context, patient-reported outcome and experience measures (PROMs and PREMs) provide critical insights into how care affects people’s lives [19]. PROMs support care that aligns with patient needs and emphasize the importance of transparency and responsiveness across health systems [18, 19]. Yet, only about one-third of European countries systematically collect patient-reported indicators—revealing a significant measurement gap [14].

Outcome-focused approaches build and foster trust and strengthen ties between health systems and communities. Transparent communication and participatory decision-making demonstrate how these methods improve care, building trust in systems that prioritize well-being and deliver tangible health benefits [5, 14, 20, 21].

Outcome measures capture the real-world impact of care, moving beyond service volumes or compliance metrics. Globally recognized indicator sets now support comparability and drive system-wide improvements. Aligning performance measurement with patient-centered frameworks enhances transparency, learning, and population health [22, 23].

Moving forward, health systems must unify measurement frameworks, financing models, and governance structures around meaningful outcomes. Standardized indicators support shared decision-making, continuous learning, benchmarking, and embedding quality across healthcare systems [17, 18, 22, 23].

### Systems perspective to quality

Quality of care is not the responsibility of individual providers alone—it is a product of an entire health system. This includes governance, financing, workforce planning, infrastructure, regulation, and accountability—all of which must work coherently if point-of-care quality is to improve. Improving quality requires a whole-system approach that ensures alignment across governance levels, guarantees equity and sustainability, and promotes a culture of continuous learning [14, 24].

This view is reinforced by systems-thinking approach, which sees health systems as complex adaptive systems [25]. Rather than functioning through top-down command or linear policy implementation, systems adapt and evolve through feedback loops, local context, and human agency. Quality improvement efforts must build on this reality and its complexity, recognizing that effective quality reform emerges from dynamic relationships across system levels—not from control alone [12, 26].

A systems approach to quality also implies policy coherence, ensuring that regulatory frameworks, compliance systems, financial incentives and accountability align and translate to improved everyday patient care. Quality must be an explicit priority with aligned objectives at the macro, meso, and micro levels, from national health strategies, action plans, and policies to health facility leadership and frontline clinical teams. Transparent outcomes measurement and reporting, on all levels, helps identify and tackle inefficient, low-value care and redirect resources to high-impact models—personalized, responsive, compassionate, and trust-building [5, 10, 27]. Systems with dedicated quality agencies, strong regulatory mechanisms, and cross-sectoral governance structures demonstrate higher levels of quality and safety performance and better health and well-being outcomes [10, 14]. Through adopting a whole-system approach, they successfully align organizational structures, policies, regulatory frameworks, and financial incentives to prioritize quality at every level of care.

A systems perspective to high-quality care must serve everyone equally by addressing systemic inequities [28]. This means designing inclusive care models that prioritize vulnerable groups and ensure that improvements in outcomes that matter are accessible to all. Cross-sector collaboration, shared networks, and clear accountability structures enhance system resilience. When quality is embedded as a core system value, healthcare is better equipped to meet diverse population needs—safely, equitably, and sustainably.

## Enablers of quality transformation

Achieving high-quality, resilient, and equitable health systems requires enablers that facilitate large-scale changes. The pillars of quality transformation define the “what,” while the enablers define the “how”—the critical mechanisms that support sustainable progress. Three key enablers create the conditions for implementing, monitoring, and scaling quality reforms while ensuring health systems remain adaptive and people-centered. To further illustrate enablers, we highlight examples of WHO’s work in the European region.

### People, leadership, and a drive for change

The workforce is the cornerstone of quality. No policy, governance structure, or digital tool can deliver quality without well-trained, engaged, and resilient health professionals [29]. While policies, governance structures, data, and digital tools co-enable reforms, it is healthcare professionals who bring quality strategies to life in practice—guided by the insights, needs, and expectations of the people and communities they serve. Embedding quality into everyday care requires aligned competencies, leadership support, and both intrinsic motivation and extrinsic incentives, alongside mechanisms for active listening, participation, and co-design. In complex adaptive and learning health systems, health professionals are not passive implementers of policy but active partners in continuously adapting and improving care, feeding lived experience and service-user perspectives into system-level governance and quality planning [29, 30]. These levers help shift behaviors, reinforce quality-aligned values, and sustain change.

Addressing persistent workforce challenges across the WHO European Region—including shortages, burnout, and inequitable distribution—requires a coordinated, cross-sectoral approach that invests in education, leadership development, governance alignment, and institutional support that aligns with workforce values and enables sustained improvement [14, 29, 30].

Leadership at all levels is vital in catalyzing transformation—from fragmented expert silos to integrated care chains and from guideline compliance to patient-centered continuous improvement. Open performance data fosters collaboration, innovation, and peer learning, encouraging professionals to reflect, adapt, and improve.

Building workforce capacity requires structured training in quality improvement, patient safety, and leadership. The “drive for change” is operationalized through initiatives such as WHO’s European Autumn Schools and Leadership Courses. Autumn schools focus on Quality of Care and Patient Safety and Child and Adolescent Mental Health, while the regional flagship European Public Health Leadership Course initiative empowers public health leaders of tomorrow (see Supplementary Table S1). These programs empower professionals to act as change agents within their health systems and thus strengthen capacity for quality improvement, leadership, and safety.

Beyond technical training, fostering engagement is crucial. Leadership pathways and role models are crucial for sustaining this culture of improvement. Quality becomes part of professional identity when embedded in career pathways, peer learning networks, and real-time feedback [30]. Quality is not what we say but what we do. Quality culture must be embedded not only in strategies and policies but in everyday practice.

### Data and transparency

Reliable, transparent, and actionable health data are essential for accountability, patient safety, and continuous quality improvement. Without robust data, systems make decisions in the dark, guided by assumptions rather than evidence. A systems approach to quality must be data-driven, embedding real-time information into decision-making to track outcomes, identify gaps, analyze variation in outcomes and guide targeted improvements. This implies strong public governance and the active involvement of governments to establish and regulate data infrastructure and standards for transparent and learning health system.

Transparency is at the center of accountability, builds public trust, and empowers stakeholders—patients, providers, and policymakers—to engage in meaningful dialogue about performance, safety, and outcomes. Trust emerges not from rhetoric but from visible, patient-centered improvements. Health systems that consistently measure and report outcomes, involve patients, and act on population needs are better positioned to earn and sustain trust. Transparent data also facilitate effective resource allocation and the spread of best practices [5, 31].

### Innovative solutions and tools

New technologies and digital tools can act as powerful enablers of health system transformation, enhancing quality, access, and patient engagement. To fulfill their potential, innovative solutions and tools must be guided by ethical principles that prioritize equity, human rights, and inclusion. If not guided by peoples’ needs and clear outcome goals, innovation can increase complexity, drive costs, worsen outcomes, and undermine sustainability. This approach to innovation prioritizes accessibility, transparency, and accountability, ensuring that all individuals—regardless of location, socioeconomic status, or health condition—benefit from technological advancements. Otherwise, without such guiding principles, innovation and digitalization risk reinforcing existing disparities and fragmentation [32, 33].

A systems approach embeds innovative digital tools across all levels of care to support clinical decisions, enable data sharing, and improve coordination. Interoperability, real-time analytics, and user-centered design are essential. However, as possibilities and uses of telehealth and artificial intelligence expand, safeguarding privacy, addressing biases, and ensuring transparency are critical to maintaining trust [31, 34]. Aligning innovative digital solutions and tools with public health goals ensures that technology advances person-centered care and leaves no one behind.

## From vision to action

### Implementing quality transformation

Transforming health systems requires more than a vision. Moving from compliance-based and volume-oriented models to those that are trust-building, patient-centered and outcome-focused calls for strong leadership, quality-focused governance, sustained investment in enablers, and alignment with broader health system goals. Critically, it demands coordinated action across people at all levels of the system.

For quality strategies to be effective, there must be clear alignment and mutual reinforcement of actions across the macro (national policy), meso (organizational), and micro (clinical) levels of the health system. Too often, quality strategies are developed without adequate input from frontline workers, facility managers, or service users [35]. Bridging this gap requires aligning governance, workforce capacity, and digital infrastructure with user engagement and actionable interventions tailored to local contexts—and supported by feedback loops. A culture of quality is essential—grounded in accountability, transparency, appropriate capacity-building, and meaningful patient engagement. Patients are not just recipients of care but advocates for quality, whose voices should influence political will, shape system priorities, and strengthen health literacy beyond clinical encounters. Quality-oriented governance frameworks embed quality as a system-wide principle, integrating accountability, engagement, equity, safety, and performance [9]. These frameworks must be supported by visible, accountable and aligned leadership at every level, from ministries to the point of care, and co-created with people they serve. They also build leadership at all levels to ensure quality improvement becomes a cultural norm, not just a policy goal.

The WHO Regional Office for Europe, through its Athens Quality of Care and Patient Safety Office, supports Member States by advancing implementation across five strategic areas (see also Supplementary Table S1):

- Advancing outcomes that matter: Supporting Member States in aligning care quality with patient- and population-level outcomes, prioritizing value over volume.
- Embedding a systems perspective to quality: Strengthening governance, policy, and financing mechanisms to integrate quality throughout the system.
- Empowering people and developing leadership: Delivering training, peer learning, and leadership development to equip and motivate professionals to lead quality reform.
- Enhancing data transparency and accountability: Strengthening data systems to enable real-time monitoring and informed decision-making.
- Innovative solutions and tools: Promoting responsible innovation, digital and telehealth strategies to improve access, efficiency, and equity.

Building on decades of work, the WHO Regional Office for Europe has intensified its support to Member States in applying these enablers in practice. While context-specific, these examples help illustrate early steps toward operationalizing the vision of high-quality, resilient, and equitable health systems. They demonstrate how leadership, accountability, and innovation can drive transformational change—and offer a foundation for embedding this agenda more systematically through the upcoming EPW2.

In Greece, the co-developed National Strategy for Quality of Care and Patient Safety (2025–2030) takes a whole-system approach to governance, clinical standards, and patient engagement. It introduces data-driven monitoring and aligns efforts across all levels of care, supported by the Greek government, the European Commission, and WHO (see Supplementary Table S1). A tailored training program for health managers fosters leadership capacity across the system. Greece has also invested in interoperable data infrastructure and developed a national quality indicators platform covering hospitals, primary care, mental health, and long-term care (see Supplementary Table S1).

In North Macedonia, a WHO-supported pilot patient safety training program demonstrated measurable gains in provider knowledge and behavior. The intervention helped integrate safety principles into medical education and clinical practice [36] (see Supplementary Table S1).

In Romania, WHO supported the development and piloting of a national set of hospital quality indicators. These indicators aim to link quality benchmarks with financial incentives, encouraging compliance with evidence-based standards and driving improvements in patient safety [37] (see Supplementary Table S1).

At the regional level, WHO developed the Telehealth Quality of Care Tool to help countries assess and benchmark the safety, quality, and user experience of telehealth services. This tool facilitates evidence-based decision-making and supports digital innovation aligned with equity and clinical effectiveness [38] (see Supplementary Table S1).

People engagement is another emerging priority. Through participatory processes, WHO has involved young people in co-designing quality standards for child and adolescent mental health services. This ensures that innovations reflect lived experience and system responsiveness [39] (see also Supplementary Table S1).

These examples highlight the importance of aligning human resources, community engagement, leadership, data transparency, and technical tools to accelerate system-wide quality transformation, by focusing on outcomes that matter.

The upcoming WHO Second European Program of Work 2026–30 will be a key vehicle for embedding this vision. Its strategic priorities—from health security to climate resilience—highlight the need for integrated, outcomes- and people-focused reform. Countries that prioritize quality of care, align governance with delivery, and focus on outcomes that matter to patients and population will be best placed to build equitable, resilient health systems fit for the future.

## Conclusion

In an era of overlapping global challenges that strain health systems and erode public trust, a new approach to quality of care is essential. This paper proposes a transformational vision that moves beyond process-oriented models, prioritizes outcomes that matter to people and embeds this perspective on quality across all levels of the system. Realizing this vision depends on three enablers: an empowered and engaged workforce, the transparent use of data to drive improvement, and innovation guided by equity and sustainability. This approach can help build more resilient, equitable, sustainable and trustworthy health systems, ensuring that high-quality care becomes the enduring foundation for the health and well-being for all.

## Supplementary Material

ckaf204_Supplementary_Data

## Data Availability

The data underlying this article will be shared on reasonable request to the corresponding author. Key points Health systems face overlapping global pressures influencing their ability to provide high-quality and safe care. This paper presents a transformational vision for quality of care, which rests on two interconnected pillars: outcomes that matter and a systems perspective on quality. Three enablers make this vision actionable: empowered and accountable leadership and workforce, transparent use of data to build trust, and responsible innovation aligned with equity. Country examples, of WHO Regional Office for Europe supporting Member States in applying these enablers, illustrate how these principles can be operationalized through governance reform, leadership training, indicator development, and digital tools.
