# Supplementary material for: Quality of care in an era of global challenges: a transformational vision for WHO European Region and beyond
Source: Eur J Public Health. 2025 Nov 5;36(Suppl 3):iii11–6. doi: 10.1093/eurpub/ckaf204 (PMC13129200; doi:10.1093/eurpub/ckaf204)
Supplement: ckaf204_Supplementary_Data [file ckaf204_supplementary_data.docx]

**Supplementary Table S1:**Implementing quality transformation: Initiatives, country support activities and publications by the WHO Regional Office for Europe and Athens Office for Quality of Care and Patient Safety

| **Activity** | **Link** |
| --- | --- |
| Taking the pulse of quality of care and patient safety in the WHO European Region report | <https://www.who.int/europe/news/item/12-12-2024-only-1-in-3-countries-in-the-who-european-region-have-an-action-plan-on-patient-safety-and-or-quality-of-care> |
| WHO Autumn Schools on Quality of Care and Patient Safety | <https://www.who.int/europe/news-room/events/item/2024/10/07/default-calendar/who-europe-launches-the-2nd-who-autumn-school-on-quality-of-care-and-patient-safety> |
| WHO Autumn Schools on Quality of Child and Adolescent Mental Health | <https://www.who.int/europe/news-room/events/item/2024/11/02/default-calendar/strengthening-quality-of-mental-health-care-for-children-and-adolescents-across-the-who-european-region> |
| WHO European Public Health Leadership Courses | <https://www.who.int/europe/news-room/events/item/2024/11/04/default-calendar/empowering-public-health-leaders-of-tomorrow--three-new-european-public-health-leadership-courses-in-kazakhstan--portugal-and-ukraine> |
| HQC-2 project (Greece) | <https://www.who.int/europe/teams/office-on-quality-of-care-patient-safety/collaborating-with-the-eu--the-hqc-2-greece-initiative> |
| Health IQ project (Greece) | <https://www.who.int/europe/news-room/events/item/2024/02/16/default-calendar/launch-of-the-health-iq-project-in-greece--improving-quality-of-care-across-the-who-european-region> |
| State of long-term care report (Greece) | <https://www.who.int/europe/publications/i/item/WHO-EURO-2024-10369-50141-75518> |
| Developing, executing and evaluating a patient-safety training programme concept for health workers (North Macedonia) | <https://www.who.int/europe/news-room/19-12-2023-who-and-north-macedonia-team-up-to-cultivate-a-safety-culture> |
| Developing and piloting a set of quality-of-care hospital indicators (Romania) | <https://www.who.int/europe/publications/i/item/WHO-EURO-2024-9873-49645-74343> |
| WHO Telehealth Quality of Care Tool | <https://www.who.int/europe/news-room/events/item/2024/03/14/default-calendar/improving-telehealth-quality-in-europe--launch-of-the-telehealth-quality-of-care-tool> |
| Youth participation in strengthening the quality of child, adolescent and youth mental health in the WHO European Region | <https://www.who.int/europe/news/item/16-11-2022-youth-demand-participation-and-change-at-first-ever-mental-health-week-in-athens> |
